# “Friendly reminder: hi! It is that time again ☺”: understanding PMTCT care text message design preferences amongst pre- and post-partum women and their male partners

**DOI:** 10.1186/s12889-021-11444-x

**Published:** 2021-08-02

**Authors:** Natabhona M. Mabachi, Melinda Brown, Catherine Wexler, Kathy Goggin, May Maloba, Dama Olungae, Brad Gautney, Sarah Finocchario-Kessler

**Affiliations:** 1 Department of Family Medicine, University of Kansas Medical Center, Mailstop 3064, 4125 Rainbow Blvd., Kansas City, KS 66160 USA; 2 Children’s Mercy Hospitals and Clinics, Health Services and Outcomes Research, Kansas City, MO USA; 3 Global Health Innovations (GHI), Nairobi, Kenya; 4 Kenya Medical Research Institute (KEMRI), Nairobi, Kenya; 5 Global Health Innovations (GHI), Dallas, Texas USA

**Keywords:** PMTCT, Principles of message design, SMS, Kenya

## Abstract

**Background:**

Prevention of mother-to-child HIV transmission (PMTCT) services in Kenya can be strengthened through the delivery of relevant and culturally appropriate SMS messages.

**Methods:**

This study reports on the results of focus groups conducted with pre and postnatal women living with HIV (5 groups, *n* = 40) and their male partners (3 groups, *n* = 33) to elicit feedback and develop messages to support HIV+ women’s adherence to ART medication, ANC appointments and a facility-based birth. The principles of message design informed message development.

**Results:**

Respondents wanted ART adherence messages that were low in verbal immediacy (ambiguous), came from an anonymous source, and were customized in timing and frequency. Unlike other studies, low message immediacy was prioritized over customization of message content. For retention, participants preferred messages with high verbal immediacy—direct appointment reminders and references to the baby—sent infrequently from a clinical source.

**Conclusion:**

Overall, participants favored content that was brief, cheerful, and emotionally appealing.

**Supplementary Information:**

The online version contains supplementary material available at 10.1186/s12889-021-11444-x.

## Introduction

Globally, 90% of pediatric HIV infections result from perinatal HIV transmission [1]. Without intervention, transmission rates of HIV can be as high as 15–45%, but with antiretroviral therapy (ART) rates can be reduced to as low as 5% during pregnancy, delivery, and breastfeeding periods [2]. Prevention of mother to child transmission of HIV (PMTCT) programs are key to providing the range of services needed for women and infants to reduce perinatal HIV infection. Such programs support ART initiation and adherence, encourage antenatal care and safe childbirth practices, and provide HIV virologic testing for infants exposed to HIV [3].

Consequently, PMTCT programs have prevented an estimated 1.4 million pediatric HIV infections globally between 2010 and 2018 [3]. In Eastern and Southern Africa, perinatal HIV infection has declined from 18% [15–25%] in 2010 to 9% [8–13%] in 2018. However, challenges engaging and retaining women in the pre- and post-natal cascade of care remain due to stigma and discrimination, limited facility resources, poor male partner support, gender inequality and economic marginalization [3, 4].

The use of mobile health (M-health) technology has proven to be an effective strategy in many settings to support engagement and retention in PMTCT services and increase early infant diagnosis (EID). Randomized trials have shown that sending short text messages is effective in improving anti-retroviral treatment (ART) adherence, viral suppression, and attendance of pre- and post-natal clinics [5–8]. The ubiquity of mobile phones in low resource settings led the United Nations to encourage the use of mobile phones as a communication strategy to provide reminders to pregnant women living with HIV and their male partners and support consistent engagement in the PMTCT cascade of care [9].

Involving participants in creating message content can be key to ensuring acceptability and appropriateness. Indeed, previous qualitative research in Kenya found that including participants in message creation alleviated concerns regarding privacy and unintentional disclosure with SMS-based reminders that could lead to marital or family discord [10]. Previous studies report that women prefer text message content to be polite and encouraging, provide informational support and reminders to take medication, attend antenatal and postnatal appointments and to engage partners [5, 7, 10].

In this study, we wanted to get a better understanding of the features of text messages including content, timing and characteristics such as tone, that would be motivating to HIV+ mothers to engage in targeted behaviors. We document the qualitative process of getting feedback and input on SMS message content with HIV+ pre/post-natal mothers and their male partners, with the aim of creating appropriate messages to encourage consistent ART adherence, engagement in antenatal clinic (ANC) appointment attendance, and a facility-based delivery.

## Methods

This qualitative study was embedded within an intervention development study in Kenya (R34MH107337), designed to develop and pilot a PMTCT component of the HIV Infant Tracking System (HITSystem) - a system-level, web-based mHealth intervention designed to improve postnatal EID outcomes. Using electronic alerts to providers and SMS text messages to mothers, the new PMTCT component of the HITSystem (v 2.0) tracks women with HIV throughout their pregnancy to support ART adherence, antenatal appointment attendance, hospital delivery, and linkage to EID services [11]. We also leveraged a supplement (R01HD076673–04 S1) piloting HITSystem 2.0 at four intervention sites to conduct this formative research. Sites were in geographically distinct counties of Kenya: Nakuru County, Mombasa County, Kakamega County, and Nandi County. Adult HIV prevalence (and ART coverage) in 2014 were 5.9% (66%) in Kakamega, 7.4% (98%) in Mombasa, 5.3% (62%) in Nakuru, and 3.7% (82%) in Nandi [12]. Study sites in Kakamega and Mombasa Counties were urban, while sites in Nandi and Nakuru Counties were rural. Qualitative data reported here were used to inform the development of the text messaging component of the HITSystem 2.0.

### Study participants

In August 2016, we conducted separate focus group discussions (FGD’s) with (1) HIV+ pregnant and/or postnatal women (5 focus groups, total of 40 participants) and (2) a subset of male partners who regularly accompanied their partners to their antenatal clinic (ANC) appointments (3 focus groups, total of 33 participants). The Nandi site had two female FGD’s and no male, while all other sites had 1 each of female and male FGD. Since these participants were already engaged with the topic of PMTCT, we felt they were best placed to provide insight on SMS content preferences. FGD’s (see Additional file 1 for questions) were conducted as part of formative work prior to any interaction with the HITSystem 2.0. Rather than individual interviews, we chose to conduct focus groups as research indicates that a group dynamic can be particularly useful for vetting and generating message content customized for the range of behaviors targeted by this intervention [13, 14].

### Message design

As this was the formative arm of the study, we developed messages prior to conducting the FGD by drawing from literature on information processing theory [15] and message design [16]. Information processing theory [15–17] posits that people who actively process a message are more likely to engage, comprehend, and positively evaluate that message; increasing the likelihood of enacting the targeted positive behavior. Literature suggests that aspects of message design [18] including: verbal immediacy (degree of directness and explicitness), message tone (cheery, serious, value free), message source (community worker, health professional, ambiguous), message length, message frequency, message timing, message visuals (emoji’s, symbols), and message language (Kiswahili, English, vernacular, sheng) could encourage active processing from HIV+ pregnant/postnatal women and their partners. With this in mind, and after consulting with our Kenyan partners (Mentor Mothers (HIV positive peer educators), project site coordinators and nurse Co-PI) who work directly with the mothers and have an understanding of the types of messages that could resonate with participants, we developed fourteen messages to pilot during the FGD’s (see Additional file 1 FGD questions)*.* Table 1 outlines the messages that were piloted and the characteristics of these messages that we felt would theoretically encourage active processing and make them more likely to succeed. Messages in bold indicate messages preferred by women while those with an asterix by men. Messages that have both indicate points of agreement between mothers and male partners.

**Table 1:** Pilot SMS texts presented to participants

Message Type (FGD type)^**b**^	English^**a**^	Message Characteristics
**ART Adherence (Women & Male)**	*“Friendly Reminder: Hi! It is that time again ☺”	Low verbal immediacy, cheery tone, positive appeal, ambiguous source
	*“Happy baby”	Low verbal immediacy,, positive appeal, ambiguous source
	*“Good health is priceless”	High verbal immediacy, positive appeal, health professional source
	“Live strong, live long!”	Low verbal immediacy,, positive appeal, ambiguous source
	**“Thank you, mama.”**	Low verbal immediacy, friendly tone, ambiguous source
**ART Adherence (Male)**	“Healthy mama = Healthy baby”	Low verbal immediacy, informative tone, emotional appeal, health professional source
**Antenatal Attendance (Women)**	*“This is a reminder that your appointment is on [date].”	High verbal immediacy (Explicit date) informative tone, health professional source
	*“Happy Baby Talk. [dd/mm/yy]”.	High verbal immediacy (Explicit date) informative tone, emotional appeal, ambiguous source
**Antenatal Attendance (Male)**	*“Your partner’s appointment is on [date]...attend with her if you can”	High verbal immediacy (Explicit date) informative/instructive tone, health professional source
	*“Father of the coming baby [date]”	High verbal immediacy (Explicit date) informative tone, ambiguous source
	*“Help her help the baby! [date]”	High verbal immediacy (Explicit date) urgent tone, emotional appeal, ambiguous source
**Facility-based delivery (Women and Male)**	*“The baby will be born this week, start panning early [date]”	High verbal immediacy, informative/instructive tone, health professional source
	“Little by little fills the container/pot”	Low verbal immediacy, positive appeal, ambiguous source
	“Investment does not rotten/get spoiled”	Low verbal immediacy, serious tone, ambiguous source

^a^All messages presented to participants in both English and Swahili.^b^Messages preferred by women are in **Bold** face font, while content preferred by male partners are noted with a “*”

### Procedures

We employed a purposive strategy recruiting pregnant and postnatal women living with HIV who were enrolled in PMTCT care at three of the planned HITSystem 2.0 intervention sites, and a subset of their male partners. Mentor mothers (women living with HIV who have completed PMTCT and employed at the hospital to provide support to PMTCT clients) who had an established relationship with female participants identified and reached out to eligible women to participate in the study. Participating women identified their eligible male partners for inclusion. Permission was sought from women with a current partner who had engaged in her PMTCT/EID care before soliciting their participation in male FGD’s.

Each FGD was scheduled on designated PMTCT clinic days and had an average of 8–12 attendees, with some participants joining late or leaving early, depending on their availability. This flexibility was deliberate to accommodate interested and eligible participants with varying clinic appointment times and work schedules who would have otherwise been unable to join. There was always a minimum of 7 participants in the focus group discussions, with 1–3 participants having to leave early or join late per focus group. While participants ability to leave early/join late may have affected the discussion, we believe the benefit of engaging participants particularly male partners who are hard to reach, the limitation created by allowing participants to be present for only part of the conversation. Focus groups were held in a quiet and private space at the study hospital and lasted about 90 min. All participants provided written informed consent and received 400 Kenya shillings (approximately 4 US dollars) in appreciation of their time.

Focus groups were conducted in Kiswahili by researchers trained in qualitative research methods. All study procedures were approved by the Institutional Review Boards at the Kenya Medical Research Institute (KEMRI) and the University of Kansas Medical Center (KUMC).

Female FGD’s were designed to help participants identify message features that were acceptable and would encourage processing, while male FGD elicited feedback on male partner involvement in PMTCT/EID and SMS content to encourage partner support pre- and post-partum. During FGD’s, facilitators first presented participants with example motivational messages designed to support appointment adherence, medication adherence, and a hospital-based delivery to elicit participant feedback. This was done to remove the pressure on participants to develop their own messages and to engender a collaborative environment. Messages were provided in both Kiswahili and English with the understanding that what is said in one language may sound very different once translated. We then reviewed each message asking participants to assist us in refining word choice, length of message, and ideal timing and frequency of messages for each targeted behavior. Once participants were feeling more comfortable with each other and the purpose of the messages, we broke them into smaller groups to generate ideas for new message content – or modify existing message content - that they would find most motivating. We reconvened as a full group to discuss new ideas and reach consensus on the strongest messaging for each targeted behavior. Concerns regarding potential unintentional disclosure of HIV status or other unintended consequences (e.g. partner suspicion of source of message) through routine text messages were explored for each specific message, recognizing pregnancy can be a particularly vulnerable period.

### Analysis

All interviews were audio recorded, translated and transcribed and an initial codebook was developed. A thematic analysis approach was be used to analyze both focus group and key informant interviews Transcripts were coded independently using Excel by two study team members for a priori and emergent themes. Transcript coding was an iterative process with cross-questioning and critique between the coders. A final codebook included themes that emerged within the principles of message design (verbal immediacy, message source, tone, appeal/incentive, length, frequency, timing, visuals and language), as well as factors that may encourage or hinder active processing. Exemplars for each theme were noted as well as the frequency and distribution of themes within the larger topic areas.

## Results

Overall, both women and their male partners anticipated high acceptability of text messages to support adherence, appointment attendance, and facility delivery. Furthermore, the desire to include their male partner or another family member in text messages regarding their care was high among pregnant and postnatal women who had disclosed their HIV status. Over half of female participants described existing support and engagement from male partners. Likewise, nearly all male partners indicated willingness to receive text messages to support their pregnant partners’ PMTCT engagement and medication adherence. Of note, although sample messages were provided to participants in both English and Kiswahili, there was no clear consensus regarding language preferences, with some focus groups preferring Kiswahili over English, based on regional and individual linguistic differences and literacy levels:

*Because if I say I want Swahili and I am not conversant with Swahili, what will I do? Or I say I want English and the next person can’t understand. Let them come and we are given options which ones we want.* (Male Partner: RV).

### Text messages to support ART adherence

While some participants described other mechanisms for remembering timely ART consumption (prompts via routines, family members, and phone alarms), and most indicated they usually took their ART medication on time, skipping doses or taking medications late was reported as a common challenge, either due to forgetfulness, fear of disclosure, or other situational challenges:

*Maybe us with babies sometimes you may get yourself busy … You find that the way you were busy you wake up and you have this and this and this to handle, before you know it time [for taking medication] is up” (Postnatal Mom: C)*

In this context, text messages could provide an additional prompt for medication adherence, indeed, participants commented as much, “Even though I always remember but that [the message] will remind me more” *(*Postnatal Mom: W*)*.

#### Supporting ART adherence: preferred message content features

Although preferences varied by participant and group, the most popular message across focus groups- “Friendly reminder: Hi it’s that time again ☺” was short in length, cheery in tone, positive in appeal, neutral in its verbal immediacy, contained a message visual, and could be from a friend. One participant commented that they liked the greeting as it seemed like a normal message to receive, “I feel [it] is good because at least someone will see maybe it’s your friend who is greeting you” (Prenatal Mom: C). Similarly, “Thank you, mama” was also popular among participants for its low immediacy (neutrality). Only a few participants indicated strong preferences for more direct health-focused messages such as “Good health is priceless” and “Live strong, live long.”

Male partner focus groups were provided the same messages with one modification: “Mama mwenye afya njema = mtoto mwenye afya” (“Healthy mama=Healthy baby”) as a prompt to support their partners. Like the women, they responded favorably to the low immediacy, friendly features of, “Friendly reminder: Hi it’s that time again ☺” with one participant highlighting the indirect nature of the message, “You see, we remind each other about [taking medication], and it [the message] has nothing to do with medication … so I am still on [like] that one” (Male Partner: C). However, unlike the women, male partners were receptive to messages such as: the “healthy mother, healthy baby” and “good health is priceless” that had greater verbal immediacy (direct), were informative in tone, had emotional appeal and could potentially be from a clinical source As one partner commented, “when she knows why she needs to take drugs, she will know the importance of the baby taking drugs and they will be healthy” (Male Partner: RV). Another participant said:

*According to that one, good health does not go to waste. That is a message you see, you will understand fast. Because good health, it’s not just those who have this condition … everyone deserves good health. So when you see that message, you will know it’s time to go take medication.* (Male Partner: C)

#### Supporting ART adherence: confidentiality through low verbal immediacy

For messages supporting adherence, confidentiality was a key consideration across all focus groups, participants responded positively to erring towards messages low in verbal immediacy and ambiguous in source. That is, messages that were indirect and neutral in content so that others using the same phone would not be able to understand the purpose of the message, “[the message content] we have chosen no one will know what they are, it’s only us” (Prenatal Moms: W).

Regardless of their personal HIV disclosure status, most participants indicated that messages should be tailored to those who had not disclosed to reduce risk, rather than creating two separate sets of messages differentiated by disclosure status, “the one who has not disclosed maybe is the one if she gets a different message it might bring problem. So if it is one [message] it also gives the sender an easy work” (Pre and Postnatal Moms: C).

Participants indicated that if messages were too vague, they could be misinterpreted raising the risk of jealousy and suspicion among intimate partners possibly leading to domestic violence or abandonment. In the context of partners being unaware of the purpose of the text messages, some participants raised concerns about the use of certain words such as “baby”, “they will ask you who is calling you baby” *(*Prenatal Mom: W*)*; emojis, “they will still ask you who is sending you [a] flower on phone” *(*Prenatal Mom: W; and greetings, “Or if you greet me and say hello, isn’t it a must he’ll [male partner] follow that number and ask, ‘And who is this and this number has no name?’ … Now this man will bring trouble.” (Pre and Postnatal Moms: C).

#### Supporting ART adherence: message timing and frequency

Preferences for the timing and frequency of adherence-related text messages varied. However, many participants preferred to receive messages twice a day (morning and evening), to correspond with their scheduled medication times, “So when I receive the SMS in the morning, I just know time to swallow medicine has come. Even in the evening when I’m sent, I just know my time has come” (Postnatal Moms: W). Given that “everyone has their own time for swallowing medicine,” some participants indicated wanting to receive a text that was tailored to their schedule indicating text messages should be sent shortly before the scheduled time, with preferred intervals ranging from 2 to 5 min in advance, “I always swallow at 8 night and 8 morning, so that time, or one minute to 8 or two minutes like this” (Pre and Postnatal Moms: C).

Some men and women indicated that over time “they will get tired” or irritated by text messages sent more than once a day (Prenatal Moms: W). Instead, those wanting only daily reminders preferred text messages sent during the time of day when they were more likely to forget. Finally, some participants expressed worry that text messages, especially frequent text messages corresponding to each scheduled dose, would compound an overreliance on phones for adherence, rather than fostering self-reliance:

*Even if it is good and I see the notion is good but it comes with negative effects. Someone can over rely on that message to come. So they can say they didn’t receive the message so they did not take their drugs … it is not all the time the phone will be charged. It can get lost or get spoilt, how will they get this message?... So what happens? Instead of helping, you have brought more problems.” (Male Partner: RV)*

Given the range of preferences for adherence timing and frequency, most participants agreed that the ability to customize frequency was important, so that individuals could select the frequency based on their needs and preferences.

### Text messages to support appointment antenatal clinic (ANC) attendance

Participants found text messages to support clinic attendance as acceptable enhancements to existing mechanisms for remembering ANC appointments. Participants described relying on clinic cards, phone calls from providers, and dwindling ART supplies in need of refilling, as cues to action for appointment attendance. However, text messages would serve as confirmation of the scheduled date, “sometimes you can look at that card and realize you looked at the date wrongly” or extra follow up for those with poor adherence, “it’s not easy you forget because the medicine itself reminds you [to attend clinic for medication refills]. Maybe now you don’t follow your own schedule of swallowing medicine. That’s when you can forget [clinic appointments]” (Prenatal Moms: C). Male partners were open to the idea of receiving reminders to support their pregnant partners, “It helps because there is forgetting, so that reminder is great.” (Male Partner: W) Even those who were hesitant to receive more frequent texts to support adherence were open to the idea of receiving appointment reminders, “Appointments [text messages] are fine because they come after a month or so, I would like that for my wife.” (Male Partner: W).

#### ANC appointment adherence: message content preferences

There was no clear consensus on which appointment reminder messages were preferred. Many participants responded favorably to messages with high verbal immediacy preferring direct appointment reminders and references to the baby - “I would pick the [“Happy baby talk”] one because it has mentioned babies there.” (Pre and Postnatal Moms: C). Some female participants indicated they would like to receive a message with a greeting and the appointment date. Unlike for adherence text messages, where confidentiality concerns were prominent, women were comfortable with the clinic as the explicit message source given that it would be public knowledge that recipients were pregnant and texts discussing antenatal care or facility-based delivery would not lead to unintentional disclosure, “so [messages mentioning] coming for clinic is not bad … because you could be going for clinic for babies … as long as you don’t mention which clinic.” (Prenatal Moms: W).

As with the women, male partners responded favorably to both the direct appointment reminder message and to the other sample messages, finding the specific mention of “baby” a positive emotional message appeal to their role as fathers to be particularly motivational. In response to the “help her help the baby” message, one male participant said, “that is most important. Because our main job is to protect the unborn child, so if we don’t remind her it will be doom” (Male Partner: W). Responding to the “father of the coming baby [date]” message, another said, “when you are told you have one that is coming, you must start preparing, there is something you are waiting for, and when you are expecting something, the heart beats fast.” (Male Partner: C).

Some male partners highlighted the importance of ensuring that text messages included accurate appointment dates to avoid additional burden on women given the challenges inherent in clinic attendance, “This [is] because if she comes, she should not be told today was not your appointment day” (Male Partner: RV). One male partner also indicated that he would prefer to receive appointment reminders in the evening, when he would be most likely to discuss the text with his pregnant partner, “That is best to come at night, around 7 when we are all at home, not when I leave” (Male Partner: W).

### Text messages to support facility based delivery

Many participants recognized the importance of a facility-based delivery to minimize the risk of HIV transmission to the infant during the delivery and early postnatal (prompt initiation of infant ART prophylaxis after delivery and linkage to EID services).

Participants discussed the need to prepare in advance to ensure a facility delivery. As such, many participants thought a reminder to prepare prior to delivery would be helpful, especially as the due date approached, “Sometimes life becomes so hard you even forget what EDD (estimated due date) is.” However, one participant indicated not wanting to receive SMS for delivery preparation for herself or her partner, because she felt it wouldn’t be needed, “It’s something you just know you will do, and if it’s your husband he’s organizing himself and he knows his wife is pregnant” (Pregnant Mom: W). Another woman thought that unengaged partners were likely to ignore the messages, “If it’s someone who is not responsible, even if you send him texts, you remind him, in words, he’ll not do” (Pregnant Mom: C). However, one male partner thought text messages for delivery would be received favorably, even by men who would not normally like to receive messages, “There are some messages some men might refuse, and there are important ones. Something that is important to you and your family you will accept, such as it’s time to give birth. If you find a message, that your wife is due to birth, you will find someone to take you to the hospital, which is also important.” (Male Partner: C).

#### Facility based delivery: message content preferences

While there was no clear consensus regarding message preference, feedback highlighted a preference for high immediacy, with more direct mention of the baby/delivery. Male partners preferred the first message (“the baby will be born this week …” ) over the second, given that it was more specific, “Number one, because “a little a little” is used in many things” (Male Partner: C).

#### Facility based delivery: message timing and frequency

Most pregnant and postnatal women indicated that they would like to receive reminders to prepare for delivery in the month prior to delivery, so that the message would come in time to prepare, but not too early so as to be disregarded, “You know again when you are told early you’ll wait” (Pregnant and New Moms: RV). Many respondents wanted to receive a text message 1 month before their EDD, while others wanted a reminder 2 weeks and 1 week prior to EDD, with several indicating they would like to receive messages as frequently as once a week, especially in the last month of their pregnancy:

*Because in the book normally we are written for EDD which is the date for delivery, but obviously, that’s not the day we are supposed to read [deliver] … So it’s good whoever is sending us message to do it two weeks before … (Prenatal Moms: W)*

However, some women wanted to receive text messages even earlier, at six or seven-month gestation to account for premature deliveries and, “because that’s when most people have complications” (Postnatal Moms: W). While male partners thought their pregnant partners could be sent more frequent messages regarding delivery preparations, a few indicated they would only want to receive text messages for themselves once or twice.

## Discussion

This formative study to develop optimal text messaging features to support PMTCT participation and retention was part of a system-level, web-based patient tracking intervention development study [11]. Previous qualitative studies have explored the acceptability and utility of using SMS messaging and understanding content preference to encourage ART adherence [19–21] and engaging in the PMTCT cascade of care [5, 7, 10, 22] In this study, we sought to better understand SMS messages that would be acceptable and encourage HIV+ pre and postnatal women and their male partners to engage in the PMTCT cascade of care including: 1) Antenatal appointment adherence, 2) ART adherence, and 3) a hospital-based delivery.

As with previous studies, participants were open to receiving text messages to help them better participate in the PMTCT cascade of care. Importantly, HIV+ women who participated in the FGD’s and had disclosed their HIV status were open to messages being sent to their male partners and conversely, partners who were already engaged in their pregnant partner’s PMTCT care were happy to be included in the communication. This is encouraging given that research indicates increased PMTCT engagement when male partners participate and provide social support [23–27].

Regarding reminders for ART medication, individual preferences and confidentiality remained critical priorities for participants. Due to confidentiality concerns and varying levels of disclosure with partners and family members, participants preferred that adherence messages were short in length, low in verbal immediacy i.e. neutral/ambiguous, cheery in tone, emotionally/positive in appeal and from a non-clinical source. Message content customization has been proposed as a way to address confidentiality issues; however, unlike previous literature this particular group of participants were more concerned with low verbal immediacy of the message, indicating [10] a preference that messages accommodate those who had not disclosed, rather than creating several custom messages. Participants also cautioned that although having an ambiguous message source can help maintain confidentiality for those who had not disclosed, it may inadvertently raise a male partner’s suspicions and jealousy regarding the message source and purpose, potentially causing an unsafe home environment. Finally, participants preferred the frequency and timing of medication reminders be once or twice a day: morning and evening, with those with a previously established ART routine more likely to prefer daily messages. Interestingly, a participant expressed that reminders could have the unintended consequence of creating a dependency on reminders instead of fostering self-reliance in the eventuality a phone is unavailable.

In regard to encouraging ANC attendance and a hospital delivery, participants were open to messages that were higher in verbal immediacy, as they felt that health appointments around pregnancy were less likely to raise HIV status disclosure concerns. Male partners liked the explicit reference to the coming baby in the message, as they saw this emotional appeal as a motivational reminder – even for those men who may not otherwise be engaged in earlier stages of the pregnancy. Overall, participants viewed these reminders as a welcome addition to clinic cards, phone calls from providers, and dwindling ART supplies in need of refilling, as cues to action for facility attendance.

The feedback and preferences of participants were used to design the final messages that were integrated into the web-based tracking intervention, HITSystem 2.0 [11]. To cut through the noise and clutter of other text messages, HITSystem 2.0 messages overall were designed per participant preferences to have an emotional/positive appeal, were brief, were low in verbal immediacy to avoid unintentional disclosure for ART messages and high in verbal immediacy for appointment and delivery reminders. Participants were given the option to opt in or out of adherence messages, given ongoing concerns about confidentiality. We stress the importance of ensuring that given the potential for stigma, violence, and ostracization SMS messages are only sent to participants who have given their express permission and feel safe receiving the messages having disclosed to their partners and/or family. Adherence messages were further customized in terms of frequency (with participants able to select daily, weekly, twice monthly, or monthly messages) and content (with participants able to select one of three messages (“ni saa”, “je hujambo”, “je, uhali gani”), which included the preferred components (“it’s time” and two friendly greeting options) of the text message most preferred by this study’s participants. Given limitations with the texting platform, the exact timing of messages could not be customized to align with individual’s dosing schedules. Appointment reminders were sent 2 days prior to each scheduled appointment and contained the text “*Tafadhali mama fika kliniki siku ya [appointment date] kwa ajili ya maudhurio ya ujauzito,tunatarajia kukuona” (*Please return to the clinic on [appointment date] for pregnancy services. We’ll be happy to see you!) Delivery support was sent 4 weeks and 2 weeks before the woman’s EDD and contained the text, “*Tuna furaha sana vile uko karibu kujifungua! Ili mtoto azaliwe na afya bora kabisa, ni vizuri ujipange kujifungulia hospitalini* (We are happy that you are close to delivering! For your baby’s best health, it is good to deliver in the hospital!) Messages for male partners have not yet been incorporated into the intervention for implementation and evaluation. Results on the overall satisfaction with the messages to HIV+ pregnant women will be reported elsewhere in a study satisfaction paper.

### Strengths and limitations

This formative study is limited by its relatively small sample size, with one focus group for each geographic region of Kenya (Western, Central, Coastal). Focus group setting can activate participants’ sense of social desirability, thus affecting their responses. Male participants were aware of their female partners HIV status and were engaged in their female partner’s PMTCT care, thus may not be representative of males less engaged in their female partner’s PMTCT care. Yet, it is a strength that the perspectives of male partners were included, as this is often missing despite the integral role of male partners in decision making during pregnancy [5, 23]. Limited demographics on participants were collected, thus, we are unable to comment on how various sociodemographic characteristics may impact preferences for text messages. The qualitative design of the study allowed for a collaborative process that provided an in-depth understanding of barriers to PMTCT participation, message content and design preferences. We also included women in different stages of their pregnancy thus eliciting a wide variety of experiences. However, we believe that the messages developed through this formative work has the best chance of being acceptable to the widest audience/all HITSystem participants. This was the formative part of a larger study and as such did not require large numbers; however, we recommend that future research that focuses on communication strategies to motivate PMTCT engagement include larger focus group or interview sample sizes that includes both HIV+ mothers and male partners. We also recommend having focus groups that include male partners who are not as engaged in PMTCT care as it may provide insight into their reasons for minimal engagement as well as the types of messaging that would be motivating to them.

## Recommendations for practice

Given our results we recommend that Kenyan PMTCT practitioners leverage the use of SMS messaging as a strategy in motivating cascade of care recommended behaviors such as medication adherence, ANC appointment attendance and hospital delivery. SMS messages can also be used for other specific programmatic goals such as social support messaging as appropriate. We stress that use of SMS messaging as a strategy must only be used with the permission of patients given the potential for unintentional disclosure with a partner or family that may lead to undesirable outcomes. For couples who have disclosed to each other SMS messages can be used to encourage male partner engagement in the PMTCT cascade of care as well as engender partner informational and emotional support that is crucial at this important time. To optimize engagement and avoid message habituation, we encourage PMTCT providers to seek the input of mothers, their partners and providers in creating messages that are the most motivating to them. Seeking input will also help account for contextual factors including geographical, language and other community differences.

## Conclusion

Our findings indicate that utilizing text messages to support engagement in PMTCT care is acceptable among women living with HIV and their partners. Engaging end users in the development of message content for key PMTCT services can optimize relevance and ensure acceptability of SMS.

## Supplementary Information

**Additional file 1.**

## Data Availability

The data that support the findings of this study are available on request from the corresponding author [NM]. The data are not publicly available as they may contain information that could compromise research participant privacy/consent.

## Abbreviations

PMTCT: Prevention of mother-to-child HIV transmission
EID: Early infant diagnosis
HITS: HIV infant tracking system
ART: Anti-retroviral treatment
SMS: Short message service
ANC: Antenatal clinic
FGD’s: Focus group discussions
