# Supplementary material for: “Friendly reminder: hi! It is that time again ☺”: understanding PMTCT care text message design preferences amongst pre- and post-partum women and their male partners
Source: BMC Public Health. 2021 Aug 2;21:1491. doi: 10.1186/s12889-021-11444-x (PMC8330020; doi:10.1186/s12889-021-11444-x)
Supplement: Supplementary file 1 — Additional file 1. [file 12889_2021_11444_MOESM1_ESM.docx]

**Additional file 1: Focus Group Questions**

**HIV+ Mothers focus group questions
Introductory Script:**

*Hello and welcome to our session today. Thank you for taking time to join our discussion on effective strategies for tailoring simple health text messages to motivate healthy behaviors during pregnancy and beyond.*

*My name is ____________and I represent the HITSystem team. Assisting me is ______________. We invited you because you have important experiences to share with us regarding your journey in the health care system as HIV positive women and mothers. Your experiences will help guide us as we develop communication messages to improve parts of your prevention of mother-to-child transmission (PMTCT) care.*

*Today we will be discussing your thoughts and opinions on the ways your health care providers can better communicate with you when it comes to: keeping appointments with your health care providers, making sure you take your medications every day, making plans for a hospital delivery of your child, and keeping track of the HIV status and health of your child once born.*

*There are no wrong answers. Please feel free to share your opinions even if they are different from what others have said. We are just as interested in positive and negative opinions and experiences*

*Before we begin there are some things that we can do to make our discussion more comfortable. Please speak up we are taping the focus group and do not want to miss any of your comments. Please feel free to address one another but try not to talk over each other. We will be on a first name basis for the discussion but in our reports there will not be any names attached to your comments. You may be assured of our utmost confidentiality.*

*My role here is to ask questions and listen. I will not be participating in the conversation, but I want you to feel free to talk to one another. Often times there are some people who talk more in these discussions than others. But it is important that we hear from each of you today because you each have different and valuable experiences. So if one of you is sharing a lot, I may ask you to let others talk. And if you are too quiet I may call on you to share more with us.*

*We’ve placed name cards on the table in front of us to help us remember each other’s names. Let’s begin. Let’s find out more about each other by going around the table. Tell us one thing you enjoy doing when you have free time. ________, let’s start with you.*

**Introductory/Overview Questions:**

1. Could you share with us some of the reasons you have for attending PMTCT clinic?
2. What are some of the challenges you have experienced with attending PMTCT clinic? *(prompt: how many of you are first time PMTCT enrollees?)*

**Transition Questions:**

1. As an HIV+ pregnant woman, what typical types of health appointments do you currently have to remember to attend?
2. Do you think it is important to attend all these appointments? Why?
3. Which appointments do you think are most helpful?

**Key Questions:**

1. When you attend/attended ANC how do/did you remember your appointments?
2. What ways do you or your health provider use to remind you to attend clinic?
3. Do your friends, family, or partner help you to attend ANC? If so, in what ways?
4. What are some of the things that make attending all of your ANC appointments challenging?

***Transition****: Let’s talk about taking HIV medications while you are pregnant.*

1. What are some of the things that made taking your medications every day challenging?
2. What ways do you or your health provider use to remind you to take your medications every day?
3. Do your friends, family, or partner help you remember to take your medications everyday? If so, in what ways?
4. What do you think about the idea of sending you text messages e.g. appointment reminders during your pregnancy? To what extent would that be helpful? Why? Why not? What other ways of communicating would work for you?
5. If you were the one sending the messages to another mother would you change the way you send the text message depending on the topic? *(Prompt: For example if you were sending a text to remind her to take her medication what types of things would you say in the text? If you were sending a text to remind her to go to ANC what would you say?)*
6. What kind of message would be off-putting to you (think about text messages you have received in the past)? *(prompt: what aspects of a message would make you annoyed or not pay attention)*
7. In front of you are three text messages reminding you to go to your ANC appointment. I’ll read each one and please describe what you like and don’t like about the message. *(prompt: How do they make you feel? Which one would you be more likely to pay attention to? Why?)*

“This is a reminder that your appointment is on [date].”

“Happy Baby Talk. [dd/mm/yy] .”

1. Now let’s come up with a couple ideas as a group. Any ideas?
2. Would it be helpful to receive text messages to remind and encourage you to take your medication every day? Why? Why not? What should messages say?
3. In front of you are sample text messages reminding you to take your medication. I’ll read each one and please describe what you like and don’t like about the message. *(Prompt: How do they make you feel? Which one would you be more likely to pay attention to? Why?)*

“Friendly Reminder: Hi! It is that time again ☺”

“Live Strong. Live Long”

“Happy Baby”
“Good health is priceless.”
“Thank you Mama.”

1. Now let’s come up with a couple ideas as a group. Any ideas?
2. Would it be helpful to have tailored messages for those who have disclosed and those who have not? Or safer to keep them all generic or have a secret code?
3. What would be the best time in the day to receive a text message reminding you to take your medication?
4. Which language would be most preferable to use?
5. Do you use emoji’s/emoticons? What do you think about us using them in texts to you?
6. How often would you like to receive medication reminders? How often is too often?
7. What are things you have done/will do to prepare yourself and your family so you could deliver in the hospital? *(Prompt: If you were sending your sister or a close friend a text to remind her to start thinking about her hospital delivery what would you say?)*
8. Now let’s come up with a couple ideas as a group for delivery planning messages. Any ideas?
9. How early should such delivery planning messages be sent? At what week gestation?
10. Are there any phrases of words we should avoid? Which words and why?
11. Do you think these ideas would be helpful to HIV+ pregnant women in other areas in Kenya and countries in East Africa?
12. Would it be helpful to have messages sent to partners, husbands, or other designated person (mother, sister, friend, etc.) of women who've disclosed their status to encourage their support in medication and appointment adherence? What should these messages say? How often should they be sent? (*Prompt: What concerns if any do you have about including partners, family members, or friends? How receptive do you feel they would be to receiving these kinds of messages?*)
13. Are there other suggestions you have on how to encourage women enrolled in PMTCT to achieve safe and HIV-free deliveries?

**Thank you so much for participating in this focus group. Please let me know if you have any final questions or comments before we conclude.**

**Male Partner Focus group questions
Introductory Script:**

*Hello and welcome to our session today. Thank you for taking time to join our discussion on males’ roles in their partner’s PMTCT care and effective strategies for tailoring simple health text messages to motivate support during your partner’s pregnancy and beyond.*

*My name is ____________and I represent the HITSystem team. Assisting me is ______________. We invited you because you have important experiences to share with us regarding your experiences as partners of HIV positive women and mothers. Your experiences will help guide us as we develop communication messages to improve parts of your prevention of mother-to-child transmission (PMTCT) care.*

*Today we will be discussing your thoughts and opinions on the ways you support your partner and challenges you’ve experienced when it comes to: keeping appointments with her health care providers, making sure she takes your medications every day, making plans for a hospital delivery of your child, and keeping track of the HIV status and health of your child once born.*

*There are no wrong answers. Please feel free to share your opinions even if they are different from what others have said. We are just as interested in positive and negative opinions and experiences*

*Before we begin there are some things that we can do to make our discussion more comfortable. Please speak up we are taping the focus group and do not want to miss any of your comments. Please feel free to address one another but try not to talk over each other. We will be on a first name basis for the discussion but in our reports there will not be any names attached to your comments. You may be assured of our utmost confidentiality.*

*My role here is to ask questions and listen. I will not be participating in the conversation, but I want you to feel free to talk to one another. Often times there are some people who talk more in these discussions than others. But it is important that we hear from each of you today because you each have different and valuable experiences. So if one of you is sharing a lot, I may ask you to let others talk. And if you are too quiet I may call on you to share more with us.*

*We’ve placed name cards on the table in front of us to help us remember each other’s names. Let’s begin. Let’s find out more about each other by going around the table. Tell us one thing you enjoy doing when you have free time. ________, let’s start with you.*

**Introductory/Overview Questions:**

1. What do you feel is the meaning and value of being a father?
2. What roles are you expected to play in the care of your child? In the care of the mother of your child?

**Perception of Women’s Need**:

1. What are a woman’s needs during pregnancy? What type of support does she need? Please think about the broader range of emotional, physical, financial, and psychological needs.
2. What are a woman’s needs when she about to deliver the baby and immediately after? What type of support does she need? Please think about the broader range of emotional, physical, financial, and psychological needs.
3. What are a woman’s needs postnatally? What type of support does she need? Please think about the broader range of emotional, physical, financial, and psychological needs.
4. Who supports each of these needs?
5. Do HIV-positive pregnant and postpartum women have any additional needs? If so, what are these? And who supports them?
6. What do you think are important things that an HIV-positive pregnant woman can do to keep herself and her infant healthy?
   1. Probe: ask about their understanding of PMTCT recommendations and early infant care.

**Male Involvement in Care**

1. How are men involved in their partner’s prenatal care? Her delivery? Her postpartum care?
2. Are there other ways you think that men would like to be involved in their partner’s care? What are these?
3. Are there other ways that you think women would want men to be involved in their care? What are these?
4. What other things could men do to support their partners during and after pregnancy?
5. What barriers do men face in being involved in their partner’s care during and after pregnancy?
6. How are men involved in the delivery of their child?
7. Who makes the decisions regarding where an infant should be delivered?
8. What are things that you have done/will do to prepare yourself and your family so you could deliver in the hospital?
9. Are there other ways that would men like to be involved in their infant’s delivery? What are these?
10. Are there other ways that you think a woman would want her partner involved in an infant’s delivery? What are these?
11. In what ways are men involved in the care of their new born infant?
12. Who makes the decisions regarding a newborn’s care?
13. Are there other ways that would men like to be involved in their newborn’s care? What are these?
14. Are there other ways that you think a woman would want her partner involved in a newborn’s care? What are these?
15. What other things could men do to support their newborn’s care?
16. What challenges do men face in being involved in their newborn’s care?

**Text Messaging**

1. Do you think that text messaging is a useful way to communicate information to men about their partner’s care?
2. What do you think about the idea of sending you text messages to help support your partner during her pregnancy (e.g. appointment reminders, PMTCT recommendations, etc.)? To what extent would that be helpful? Why? Why not? What other ways of communicating would work for you?
3. If you were the one sending the messages to another soon-to-be father would you change the way you send the text message depending on the topic? (Prompt: For example if you were sending a text for him to remind his partner to take her medication what types of things would you say in the text? If you were sending a text for him to remind his partner to go to ANC what would you say?)
4. What kind of message would be off-putting to you (think about text messages you have received in the past)? (prompt: what aspects of a message would make you annoyed or not pay attention)

*Appointment Reminders*

1. Would it be helpful to receive messages reminding you of your partner’s upcoming appointments? Why/why not?
2. In front of you are three text messages reminding you of your partner’s ANC appointment and encouraging you to attend with her. I’ll read each one and please describe what you like and don’t like about the message. (prompt: How do they make you feel? Which one would you be more likely to pay attention to? Why?)

“Your partner’s appointment is on [date] –attend with her if you can!”

“Proud father-to-be! [dd/mm/yy].”

“Help her help Baby! [dd/mm/yy]”

1. Now let’s come up with a couple ideas as a group. Any ideas?
2. What considerations for the timing and frequency of these messages do you think are important? How often would you like to receive these messages? What time of day is best for them to be sent? How far in advance do they need to be sent?

*Medication Adherence*

1. Would it be helpful to receive text messages encouraging you to support your partner’s medication adherence? Why? Why not? What should messages say?
2. In front of you are sample text messages reminding you to take your medication. I’ll read each one and please describe what you like and don’t like about the message. *(Prompt: How do they make you feel? Which one would you be more likely to pay attention to? Why?)*

“Friendly Reminder: Hi! It is that time again ☺”

“Live Strong. Live Long”
“Good health is priceless.”
“Healthy mama = health baby”

1. Now let’s come up with a couple ideas as a group. Any ideas?
2. What would be the best time in the day to receive a text message encouraging you to support your partner’s medication adherence? What other considerations for timing/frequency do you think are important?

*Hospital Delivery*

1. Would it be helpful to receive text messages encouraging you to prepare for a hospital delivery? Why? Why not? What should messages say?
2. In front of you are sample text messages reminding you to take your medication. I’ll read each one and please describe what you like and don’t like about the message. (Prompt: *How do they make you feel? Which one would you be more likely to pay attention to? Why?)*

“Baby comes in X weeks! Start planning early!”

“A penny saved is a penny earned”

1. Now let’s come up with a couple ideas as a group for delivery planning messages. Any ideas?
2. How early should such delivery planning messages be sent? At what week gestation?

**General Questions/Wrap up**

1. Which language would be most preferable to use?
2. Do you use emoji’s/emoticons? What do you think about us using them in texts to you?
3. Are there any phrases of words we should avoid? Which words and why?
4. Do you think these ideas would be helpful to HIV+ pregnant women in other areas in Kenya and countries in East Africa?
5. Are there other suggestions you have on how to encourage men to support their partners PMTCT care to achieve safe and HIV-free deliveries?
